# Acceptability of the BATHE technique amongst GPs and frequently attending patients in primary care: a nested qualitative study

**DOI:** 10.1186/s12875-019-1011-y

**Published:** 2019-09-03

**Authors:** Clare Thomas, Helen Cramer, Sue Jackson, David Kessler, Chris Metcalfe, Charlie Record, Rebecca K. Barnes

**Affiliations:** 1 Centre for Academic Primary Care, Population Health Sciences, Bristol Medical School, University of Bristol, Bristol, UK; 2 Centre for Academic Primary Care, Population Health Sciences, Bristol Medical School, University of Bristol, Canynge Hall, 39 Whatley Road, Bristol, BS8 2PS UK; 3 Psychology Dept, University of the West of England, Bristol, UK; 4 Bristol Randomised Trials Collaboration (BRTC), Population Health Sciences, Bristol Medical School, University of Bristol, Bristol, UK; 5 Frome Valley Medical Centre, 2 Court Rd, Frampton Cotterell, Bristol, BS36 2DE UK

**Keywords:** Primary care, Person-centred care, BATHE, Frequent attenders, Qualitative methods

## Abstract

**Background:**

BATHE is a brief psychosocial intervention designed for physician use in patient consultations. The technique has gained some international recognition, but there is currently limited research evidence to demonstrate its acceptability and benefits to patient care. We conducted a pilot cluster randomised controlled trial and feasibility study to explore the use of BATHE as a key component of a person-focused intervention to improve the care of frequent attending patients in UK primary care.

**Methods:**

A nested qualitative interview study conducted within a pilot trial. The trial took place in six general practices in the South West of England. Eligible patients had been identified as being in the top 3% of attenders in the previous 12 months. General practitioners (GPs) were trained to use BATHE during a one-hour initial training session, and two top-up trainings which included feedback on implementation fidelity. GPs were asked to use BATHE with their study patients for a period of 12 months. 34 GPs were trained and documented using BATHE in a total of 577 consultations with eligible patients during the intervention period. At the end of the intervention period, GPs and study patients from the intervention practices were invited to take part in an interview. Interviews were semi-structured, audio-recorded and transcribed. Thematic analysis was used.

**Results:**

Eleven GPs and 16 patients took part in post-intervention interviews. Benefits of using BATHE included making consultations more person-centred, challenging assumptions that the GP knew what was going on for the patient and their main concerns, and supporting self-management. Difficulties reported included changing existing consultation habits, identifying appropriate consultations in which to use BATHE, and organisational constraints.

**Conclusions:**

The study suggests that using BATHE is both acceptable and beneficial but also highlighted some of the difficulties GPs had incorporating BATHE into routine practice. Strategies to reduce these difficulties are needed before the extent of the potential benefits of BATHE can be fully assessed.

**Trial registration:**

ISRCTN62939408 Prospectively registered on 24/06/2015.

## Introduction

The UK Royal College of General Practitioners (RCGP) curriculum guidelines on the core capabilities and competencies of being a General Practitioner (GP) states that as a GP you should “adopt a person-centered approach in dealing with your patients and their problems, in the context of their circumstances” [1]. Such consultations are also preferred by patients, and in particular those with psychosocial problems, worry and high levels of attendance [2]. However, GPs are dealing with unprecedented levels of demand on time and resources [3]. Under these circumstances exploring and addressing the wider context of people’s health concerns is challenging.

The Footprints in Primary Care Study, was a pilot cluster randomised controlled trial (RCT) and feasibility study exploring an intervention for frequent attenders in primary care [4]. The idea for the study originated in a primary care practice in South West England who felt that improvements could be made in the care of patients who attend most often. The practice developed a RCGP award-winning intervention consisting of several components including training GPs to use the BATHE consultation technique. BATHE was developed by Stuart and Leiberman in the United States of America as an ‘aide memoire’ for family physician trainees to encourage routine consideration of the psychosocial aspects of patients’ presenting complaints [5]. BATHE is an acronym for five different elements; **B**ackground, **A**ffect, **T**rouble, **H**andling, and **E**mpathy. The first four elements are a series of linked questions to be asked towards the end of history-taking for the patient’s presenting complaint and the final element is an empathetic statement (Table 1). As such the technique can be used to “connect meaningfully with the patients, screen for mental health problems, and empower patients to handle many aspects of their life in a more constructive way...[and] can be accomplished in about 1 minute” (Leiberman and Stuart, 1999, pg 39) [6].

**Table 1:** The five elements of the BATHE technique and their related questions [5]

B = Background	What is going on in your life?
A = Affect	How do you feel about that?
T = Trouble	What about the situation troubles you the most?
H = Handling	How are you handling that?
E = Empathy	Closing Statement. That must be difficult for you (or an appropriate alternative of a similar nature).

The BATHE technique has potential to support the delivery of person-centred care in a time restricted context. It is therefore a good fit for a primary care intervention to improve the management of frequently attending patients. The technique aims to help health professionals explore the wider context to the patients’ health problems, identify unmet needs and enable the provision of appropriate treatment or support for self-management.

BATHE has gained popularity around the world. The book describing the technique first published in 1986, is currently in its 6th edition [5], and has been translated into several different languages. Furthermore, the benefits of using BATHE have been the subject of a number of editorials and opinion pieces in the medical press in both the UK and USA [7–10]. Despite this global interest, there has been very little primary research exploring the benefits of its use. Two relatively small scale studies, one in the USA and one in Korea, demonstrated increased levels of satisfaction amongst patients where BATHE had been used [11, 12]. In addition, a RCT of the use of BATHE with diabetic patients, conducted in Turkey, demonstrated an increase in the Diabetes Enablement Scale [13]. Our study aims to expand the current evidence-base for BATHE by exploring the acceptability of its use amongst GPs and frequently attending patients in UK primary care.

## Methods

### Design

This was a nested qualitative interview study conducted as part of a mixed methods process evaluation in our pilot cluster RCT. Qualitative methods were employed in order to gain a deeper understanding of participant experiences and to aid in the interpretation of the quantitative analysis of clinical and intervention fidelity outcomes in the pilot trial.

### Setting

The pilot RCT involved six general practices in the South West of England. Practice eligibility criteria were; having medium to large patient list size and the organisational capacity to participate (with at least three participating GPs). Practices meeting these criteria were identified by the Clinical Research Network West of England and invited to participate. Recruited practices were randomly assigned to either intervention or control in a 2:1 ratio.

### Intervention

Use of the BATHE technique was one element of a multi-component intervention which also aimed to increase continuity of care by assigning patients a named GP and to increase use of telephone consultations wherever appropriate. To support the use of BATHE, GPs in intervention practices were invited to attend in-house training. The training consisted of an initial session at the start of the study, and two top up trainings at around 4 and 7 months. Each session was an hour in length. The initial training included an introduction to the BATHE technique and its underlying principles (for further details about the training see Barnes et al. (2019) [4]). GPs were also trained to initiate BATHE towards the end of history-taking and encouraged to adhere to the original question wording (see Table 1) as much as possible. Practices were each given two copies of the BATHE book ‘*The Fifteen Minute Hour, 5th edition*’ [14] and GPs were given a small prompt card to remind them of the five BATHE components to place in their consulting rooms. They were encouraged to practice using the technique ahead of the intervention ‘go live’ date. During the 12-month intervention period, GPs were asked to incorporate BATHE into all consultations (where appropriate) with study patients and to document its use in the patient’s electronic health record for audit purposes. A pop-up message was added to the records of all study patients reminding GPs of their status. Anonymised data were collected from practice records every 6 weeks so that the number of consultations with study patients and the extent to which BATHE was being used by individual GPs and practices could be monitored by the research team. Two self-selecting GPs from each practice were also asked to supply a small sample of recordings of BATHE consultations with consenting study patients. The audit information, along with insights gained from reviewing the recordings of GPs actually using BATHE [15], were fed back to the GPs at the top up training sessions.

The results of the pilot trial are reported in full elsewhere (4). In summary, 34 GPs attended at least one BATHE training session between July–October 2015. Over the subsequent 12 month intervention period, BATHE use was recorded in 9.7% (*n* = 577) of all consultations with eligible patients (range across intervention practices 7.2–19.2%). 50.1% (*n* = 207) of all eligible patients in intervention practices were exposed to BATHE one or more times (range across practices 36.4–84.1%).

### Participants

Prior to randomisation, practices were instructed to search their records for all registered patients aged 18 or over who were in the top 3% of attenders over the last 12 months. This list of potentially eligible patients was then reviewed by GPs in each practice to remove patients meeting the study exclusion criteria (see Barnes et al. (2019) for details [4]). All eligible patients were invited by post to participate in completing questionnaires for the RCT and asked to indicate whether they would be happy to be approached at a later date for interview. In addition patients in the intervention practices were informed that the GPs in their practice would be receiving extra training in consultation skills, but were not provided with any detail about the BATHE technique.

Participants invited to take part in the nested qualitative interview study were purposively sampled to include: GPs and patients from all four intervention practices, GPs with a range of levels of engagement with BATHE, and patients with a range of attendance rates and level of exposure to BATHE. GPs were invited to take part by email, either directly or via the practice manager, information sheets were provided, and written consent obtained. Patients were sampled from those taking part in the RCT who had given consent to be approached about an interview. They were contacted by telephone to be invited, and a written information sheet and consent form was then sent by post or email to those agreeing to take part.

### Data collection and analysis

The GP and patient interviews were one component of a mixed-methods process evaluation which accompanied the pilot RCT. This evaluation also included observations made during practice visits and training sessions, ethnographic observation of appointment-making in intervention practices, audit data from practice records, recordings of BATHE consultations, and interviews with practice managers and reception staff.

The GP and patient interviews took place at the end of the 12-month intervention period, between October 2016 and January 2017. They were semi-structured using separate topic guides for GPs and patients. The topic guides were developed by the research team prior to the interviews and included items to address the objectives of the feasibility study, and the accompanying process evaluation. GP interviews were conducted either face-to-face or by telephone by HC. Face-to-face interviews took place at the GPs’ place of work. Patient interviews were conducted by telephone by CT and HC. All interviews were audio-recorded and transcribed verbatim. Initial independent coding by HC, RB and CT of a subset of transcripts was guided by a framework relating to the aims of the process evaluation. The framework included broad themes such as the feasibility and acceptability of recruitment procedures, GP and reception staff training and all the components of the intervention. Following discussion, the final agreed coding framework was refined and applied to the whole data set supported by NVIVo software (QSR International, Melborne, Australia). In order to generate the findings reported in this paper data coded under the broad theme “acceptability of BATHE” was analysed in more depth using thematic analysis [16]. This analytic method involves familiarising yourself with the data, coding interesting features of the data systematically, collating codes into themes and checking back that the themes work with the coded data and the dataset as a whole, and finally refining, naming and defining themes so they can be reported.

## Results

All 11 GPs approached to take part in the interviews agreed to take part. Eighteen patients were selected for invitation. Sixteen were interviewed, one could not be contacted at the invitation stage, the other agreed to take part but then could not be contacted to conduct the telephone interview. Characteristics of the participants are detailed in Table 2. GP interviews were 30–56 mins (mean 38.4 mins) in length, patient interviews were 9–40 mins (19.7 mins) in length. Very few patients recalled their GPs using the BATHE questions so what follows is based largely on GP interview data, although patient views are included where they are available. The findings concerning the acceptability of BATHE divide into two broad themes; the benefits of using BATHE and the difficulties with using BATHE. These broad themes contained a number of subthemes (see Table 3) described below and illustrated with verbatim quotes.

**Table 2:** Summary of Interview Participant Characteristics

GP Interviews (*n* = 11)		
Practice (n)	Practice 2	3
	Practice 3	2
	Practice 4	3
	Practice 5	3
Type of interview	Face-to-face	7
	Telephone	4
Gender (n)	Male	3
	Female	8
GP use of BATHE	% of consultations where code for BATHE was added to the patient electronic record (mean (range))	18.5 (3.2–43.0)
Patient Interviews (*n* = 16)		
Practice (n)	Practice 2	5
	Practice 3	2
	Practice 4	5
	Practice 5	4
Gender (n)	Male	5
	Female	11
Age	Age in years (range)	25–88
Exposure to BATHE	% of consultations where BATHE was used (mean (range))	18.0 (0–60.0)

**Table 3:** Summary of themes and sub-themes arising from the analysis

Theme	Sub-themes
Benefits of using BATHE	Supporting contained person-centred consultations
	Challenging assumptions
	Providing new insights about patient’s primary concerns
	Validating experiences and feelings
	Supporting self-management
Difficulties with using BATHE	Fit with habitual consultation styles
	Interpretations of the appropriate use of BATHE
	Language and cultural difficulties
	Organisational constraints
	Knowing what to do next

### Benefits of using BATHE

#### Supporting contained person-centred consultations

Many of the GPs had a positive view of the BATHE technique and could see its benefits in making consultations more person-centred, in particular by increasing understanding of the wider context to patient’s problems.

*I think it is good for patient-centric consulting. (Practice 4, GP3)*

*My thoughts are I understood them better. I understood their context and their problems better. (Practice 4, GP6)*

The idea that consultations should be more personalised was also identified as important in patients’ accounts. Some patients felt they had noticed an improvement during the study period.

*I think that’s a personalising of things and that’s making a personal contact, that’s a recognition of you, that’s very important. (Practice 4, patient 408)*

*I would say that more recently, over the last year, they have been more caring. I suppose that’s the word. Taken more interest. (Practice 2, patient 204)*

GPs also felt BATHE had helped to improve their relationships with their frequently attending patients, who otherwise may have been a source of stress or frustration.

*I think what it does do, though, is it gives you a really nice tool for dealing with patients, where you are thinking, “Why has this person come back in? I just saw her a couple of weeks ago.” I suppose you can become either frustrated or defensive or annoyed, or something like that. I think what the BATHE gives you is something to say … let’s focus on the underlying emotional issues and the self-empowerment bits of this. So, let’s use this as a therapeutic intervention rather than just a waste of my time. (Practice 4, GP3)*

*I feel my relationship with him has improved by using BATHE over the last year … I'm perhaps a bit more empathetic towards his situation than I was prior to using it because a year ago I was possibly somewhat frustrated with him, whereas now I think we get on a lot better in terms of our rapport. (Practice 5, GP4)*

The structured nature of the technique was reported as helpful for GPs to focus and contain their discussions with patients, who often come with multiple and complex problems.

*I felt a bit more in control of the situation using it. Rather than feeling that the patient was talking and it was going onto every topic because the questions are quite specific it encourages the patient to give a fairly compact answer. Without using the technique I would be stuck on hearing on what is going on in your life, not knowing what to do with it. (Practice 4, GP8)*

#### Challenging assumptions

GPs acknowledged that due to the regularity of contact with frequent attenders, it was easy to assume they knew what was going on for the patient. Using BATHE helped them to challenge these assumptions and create space for patients to disclose new or unanticipated information.

*I suppose for instance, “What is going on in your life?” Then actually asking the patient how they feel about that it might often have been a different answer to what I was expecting. The problem that I thought I could make assumptions about how that was affecting them or making them feel, but it might have been quite wrong. (Practice 4, GP8)*

#### Providing new insights about patient primary concerns

A number of the GPs could recall specific instances where using BATHE elicited new insights about what was most important to the patient, which may extend beyond their medical complaint.

*It's a good way of keeping in touch with the things that are important to people. Things that are going on in their lives. That aren't necessarily medical, but may impact on their medical symptoms. (Practice 4, GP6)*

The following accounts of disclosures by a patient with chronic obstructive pulmonary disease (COPD) and a woman experiencing domestic violence are powerful examples of the insights gained.

*Just one man in particular, I remember, he kept coming back with COPD and kept getting lots of antibiotics and steroids and it wasn’t totally clear as to whether he actually needed them, but he’d keep coming in and on calls, and he said, “My biggest worry is that I wake up in the middle of the night, and I’m going to die on my own, when I’m getting breathless. There’ll be nobody with me.” Then we could talk through that and discuss it and get him extra support, discuss his feelings and discuss about COPD eventually being a terminal illness and helping him to cope with that possibility as well because there’s no point in denying it, and that really helped him. Now he doesn’t come back nearly as much, so that was a more striking example. (Practice 5, GP6)*

*I think there was an example of a woman who was having marital problems and they sound pretty awful. The question was, “What troubles you about this the most?” We were expecting her to say the domestic abuse and all the awful things like that and she was thinking of leaving him. Actually her answer was, “What troubles me the most is I always wanted my children to not grow up in a broken marriage.” She was more worried about the divorce than what was going on for her. That was quite surprising that she was going to try to maintain this relationship against all odds. We had been working on the fact that she was probably going to have to leave him because it wasn’t safe. (Practice 5, GP3)*

As well as eliciting new disclosures, the ‘T’ question, “What is troubling you the most?” was particularly valuable in helping to focus the consultation and guide the support offered.

*That was the question I liked the most. ‘What really troubles you about that,’ and then … for them think about the five problems they’ve brought or the whole melee of things they’ve just thrown at you and just think, “Which is the one thing?” … [It] enabled me to think, “Well, that’s the one we’re going to try and concentrate on, hone down on and think about.” Sometimes you can do something and sometimes you can’t. (Practice 2, GP8)*

#### Validating experiences and feelings

GPs acknowledged the benefit of the BATHE questions in ensuring patients felt heard and understood and that their feelings were validated.

*I think those perhaps are some of the most important bits of it. Someone being heard. Yes. You’re understanding what it is that is affecting them the most, and then you’re connecting with that. (Practice 4, GP3)*

*Maybe they have left feeling a little bit empowered or they are validated that they have been able to express how they felt about all these awful things going on. Maybe I didn’t worry so much that there was more I should have been doing. What was bothering them was they felt angry, so we could talk about that a little bit, but without me having to fix all these multiple different issues. (Practice 4, GP8)*

However, patients’ views of this benefit were mixed. Whilst one patient felt being provided a space within the consultation to express difficult feelings and have them validated was very valuable, another was uncomfortable with being asked about his feelings.

*For somebody who can’t take many of the drugs I’ve been prescribed over the years, it can be very lonely and very isolating … It would occasionally be helpful to say to the GP, “I’ve been as pissed off as hell and as down as hell the last few weeks because of this.” There isn’t time anymore to do that or say that. (Practice 4, patient 409)*

*Patient: Well, there was one time I went to see her. She did ask me how I was feeling. So, that was unusual. She’d never asked that. Do you know what I mean? In terms of emotional, and stuff like that … .*

*Researcher: Yes, and when she asked you how you were feeling, how did you find..?*

*Patient: I didn’t want to get into it...I don’t even want to think about it. If I think about it, then I get depressed … So it’s better, the way I look at it, block it out, don’t think about it, just get on with it.*

*(Practice 3, Patient 309)*

#### Supporting self-management

Some GPs also felt BATHE was helpful in supporting patients to think of their own solutions to their problems, and therefore encourage greater self-management.

*You’re showing that you’ve understood that, but you’re also reinforcing their ability to cope themselves. (Practice 4, GP3)*

*I do recall occasions using those questions and finding that quite a useful way of improving the direction of the consultation, helping the patient come up with the ideas and solutions and feel a bit more self-sufficient. (Practice 4, GP8)*

### Difficulties with using BATHE

#### Fit with habitual consultation styles

Whilst many GPs were positive about BATHE from the outset and willing to give it a go, others were more reticent, with their initial response being “we do this already”.

*I think we were doing that a bit before, not as prescriptive as the BATHE. I don’t feel it has been a revolutionary change for me I am afraid. (Practice 3, GP2)*

*I think the thing is that I have got a reasonably ingrained consultation style and structure and it pretty much incorporates the BATHE technique anyway. (Practice 5, GP3)*

Some GPs felt that the wording of the questions was awkward and artificial. They also reported finding it difficult to change their habits by incorporating BATHE into their ‘default’ consultation style.

*My initial reaction is that was going to sound quite contrived … if you keep asking the same thing with the same people that it could almost become like a joke. If ever any of them knew each other, they probably don’t and were to talk to each other, “Did she say, ‘What is troubling you about this the most?’ That is what she always says.” (Practice 5, GP3)*

*It felt like it was another thing to do in the consultation rather than as part of the consultation. (Practice 2, GP11)*

*Yes, very, very clunky. And it took a long while actually, to get used to it … So switching between the styles, I found quite difficult. And it very much slowed me down in terms of getting used to it and doing it. (Practice 4, GP6)*

*I found that possibly because I have been a GP for so many years it was quite hard to change habits and to get used to asking the questions. (Practice 4, GP8)*

#### Interpretations of the appropriate use of BATHE

There was variation between GPs in their assessment of when it was appropriate to use BATHE. Examples of the contexts in which some GPs felt it was not appropriate included during medication reviews or test result discussions, or when the patient was reporting a straightforward physical complaint.

*If somebody has come to review their hypertensive medication, BATHE doesn't quite fit it so well, does it? Because that's just a very bread and butter, run of the mill, take the car in for an MOT and a service sort of consultation … I did try it in some, and found that it didn't sit quite so comfortably, and didn't feel quite right. (Practice 2, GP5)*

*It doesn't work well when somebody's got a very straightforward problem that's completely relevant, and it's completely right that they came, and it's a very physical thing, and it's a new problem. (Practice 2, GP5)*

Some GPs were also unconvinced about the benefits of using BATHE repeatedly when patients were attending regularly.

*I was seeing her quite a lot, so I didn’t feel like I could BATHE her everyone time, it just felt really contrived (Practice 2, GP8)*

*I think there's value in that style of questions in terms of the information you get out and the way it can help you communicate and develop a joint agreement about where you're going, so you're using it once or occasionally. Using it every week or every two weeks or every three weeks, for me, I'm not convinced that would drive down re-attendance rates. Sorry. (Practice 5, GP4)*

Similar sentiments were expressed by one patient about how necessary or appropriate BATHE would be if you already had a good relationship with your GP or you were attending to discuss a simple physical complaint.

*I have a very good relationship with him so if I’ve got a problem I will tell him about it. I don’t know that he would see it necessary to cross-question me, if you like, about anything like that … If I saw someone else, as I say, it would probably only be for something superficial like a sore throat that’s gone on for a while or whatever so I wouldn’t expect really that they would want to use their time to ask me questions like that. I mean, I think they would just want to deal with what I was going into the surgery about. They only have ten minutes for their appointment anyway, I wouldn’t think that they would find it necessary to go any deeper into things. (Practice 2, Patient 220)*

#### Language and cultural difficulties

One surgery involved with the study had a high proportion of non-English speaking patients and the use of interpreters was common. Whilst some GPs felt it was possible to use BATHE in this context, challenges were also highlighted in terms of the additional time required and uncertainties around cultural appropriateness of the questions.

*The group for whom English is not their first language, it felt quite difficult as a practitioner to fit that into the consultation because time constraints are even more stretched. (Practice 5, GP4)*

*There isn’t the same immediate level of understanding when you say to somebody, “What troubles you most about that? … Most British nationals, English mother tongue, will know or suspect that you are getting at maybe the emotional impact and psychological impact when you ask that question. Whereas for a lot of Somalis the word stress still isn’t in their vocabulary. They are a bit more inclined to be like, “I have got the illness. You are the doctor, you tell me.” (Practice 5, GP3)*

#### Organisational constraints

Some GPs felt being under the time pressure of the 10 min consultation made it difficult to use BATHE, and therefore some reported choosing not to use BATHE if they were already running late, or when they saw an opportunity to catch up time by not adding it into a straightforward consultations. It was also noted by one GP that she wouldn’t use it if she was the rostered duty doctor.

*I didn’t use it to begin with because I think it was very, very busy … when … fitting in extra patients and you’ve got a zillion phone calls, you know, it just didn’t feel like a priority. (Practice 3, GP3)*

*If they had come in about a slight bruise on their big toe and then you went and asked all those questions it could easily allow them to talk about all sorts of other things. If I had just been very closed, dealing with that one problem, getting them out of the door would have been much quicker. It could open a bit of a can of worms. (Practice 5, GP3)*

GPs reported some difficulties with remembering to use BATHE. They acknowledged that the pop-up messages aimed at reminding them were often ineffective because these types of message were already used for too many other reasons.

*I think one of the biggest challenges is the alert. I think we probably missed quite a lot of opportunities to do the BATHE technique, because there are so many alerts in general practice now. (Practice 2, GP5)*

#### Knowing what to do next

Some GPs expressed concerns about what they would do with the information elicited when using the BATHE questions, particularly if they highlight wider social problems which the GP felt unable to resolve. One respondent raised the question of whether this type of discussion was therefore a good use of a GPs time.

*Part of the problem is, you find out all the stuff about somebody’s life, and frequent attenders and their mood, and things like that, and it’s knowing what to do with that information. (Practice 5, GP6)*

*I think certain patients, when you know that they might have social reasons for attending and you’re trying to do the consultation, you know, someone’s come in for a non-medical reason and you’re running very late, it’s almost sort of you’d want to prioritise people that are sick and trying to get through the consultation more quickly rather than sitting there and sort of discussing sort of social factors that you might not really be able to help or assist with. (Practice 3, GP3)*

## Discussion

This nested interview study explored GP and patient views on the acceptability of the BATHE technique as part of the process evaluation within a pilot RCT of an intervention to improve care of frequently attending patients. Many of the GPs interviewed reported feeling positive about using BATHE and were able to recall examples where it had been beneficial in delivering more person-focused consultations, and in improving their relationship with frequently attending patients. Patients also valued these benefits. GPs found BATHE a useful tool for challenging assumptions such that they already know the patient’s situation and main concerns, for validating feelings and for supporting self-management.

The interviews also revealed several difficulties with using BATHE. These included difficulties in changing consultation habits and learning a new technique, in knowing what types of consultations it was appropriate to use BATHE in, organisational constraints and language and cultural difficulties. Insights into these difficulties are valuable, and when combined with other findings from the pilot RCT and process evaluation [4, 15], help us to understand our quantitative findings regarding overall use of BATHE. Whilst feedback from study GPs suggested there was some under-reporting of its use, the extent of BATHE use was still lower than we might have hoped. However, there is currently no established threshold for the extent to which BATHE would need to be applied in order for benefits to be realised and given that GPs felt there were some circumstances when using BATHE would not be appropriate, it may be that focus on the proportion of individuals who were exposed at least once is a more salient measure of intervention delivery than the proportion of all consultations.

This work highlights several ways in which training and procedures could be improved in order to increase the use of BATHE in future research or practice contexts. For example, the benefits of using BATHE can now be illustrated by quoting the real-world examples given by GPs in our study, which may convince other GPs to give BATHE a try and not to prejudge it as no different to usual care. Further clarity could also be provided about the appropriate contexts in which BATHE can be used, for example, by discussing its potential for uncovering important underlying issues such as mental health difficulties or domestic violence for someone who is repeatedly consulting with seemingly straightforward physical complaints. In a separate analysis of consultations recordings [15], we highlighted the importance of asking the BATHE questions at the appropriate time within the consultation. We found that GPs were often using BATHE too early in the consultation, which resulted in the questions landing ‘awkwardly’ and failing to elicit the intended information. This in turn may have contributed to the feeling that the questions were contrived or unnatural, which understandably affected GPs willingness to continue using BATHE. Following the top-up training sessions where some of the learning points described above were shared, BATHE rates were seen to increase amongst the GPs who attended the training. In future research we would also need to consider alternative ways to provide point of care prompts for GPs, given that pop-ups on the patient records did not seem to function well as reminders.

The strength of this study is that, to our knowledge, it is the only qualitative study exploring acceptability of BATHE to both doctors and patients. It is therefore an important addition to the body of peer-reviewed literature on BATHE, of which there is a marked paucity given that the technique is internationally recognised particularly in the medical education context. Furthermore, it should be noted that this limited availability of evidence extends to other commonly taught person-centred consultation techniques such as the ideas, concerns, expectations model [17].

The study has some limitations. Whilst we aimed to sample GPs with different levels of engagement with using BATHE, we acknowledge that the practices involved took a favourable view on the aims of the study, which may affect the generalisability of the findings. Furthermore, the patient perspectives gathered in this study were more limited than the GP accounts. Most of the patients interviewed did not have a specific recollection of BATHE being used in their consultations. Whilst this limited the feedback they were able to give, it may also suggest that patients didn’t find receiving BATHE as awkward and unnatural as the GPs sometimes felt when delivering it. The views expressed by patients that BATHE had potential for improving their relationship with their doctor was echoed in subsequent discussion between the research team and the study patient and public involvement (PPI) group. Further work with patients would be beneficial to explore their perspective in more depth. A robust evaluation of the effectiveness of BATHE as a brief psychosocial intervention would also expand on the existing literature showing benefits for patients in satisfaction and psychosocial self-efficacy [11–13].

## Conclusions

The findings presented in this paper, in combination with other evidence collected during the wider feasibility study [4, 15], suggests BATHE is acceptable and potentially beneficial when used in primary care to support the delivery of person-centred care within a time-constrained consultation. The study also highlighted some of the difficulties GPs found incorporating BATHE into routine practice. Strategies to reduce these difficulties are needed to increase implementation fidelity, which would then allow a larger scale evaluation of the benefits of using BATHE for both patients and healthcare practitioners.

## Data Availability

Anonymised versions of the interview data reported in this paper are available from the corresponding author on reasonable request.

## Abbreviations

BRTC: Bristol Randomised Trials Collaboration
CTU: Clinical Trials Unit
GP: General Practitioner
NHS: National Health Service
NIHR: National Institute for Health Research
RCGP: Royal College of General Practitioners
RCT: Randomised controlled trial
RfPB: Research for Patient Benefit (RfPB)
UK: United Kingdom
UKCRC: United Kingdom Clinical Research Collaboration
USA: United States of America
